# Fluid therapy in critical illness

**DOI:** 10.1186/2046-7648-3-16

**Published:** 2014-09-29

**Authors:** Mark R Edwards, Michael G Mythen

**Affiliations:** 1 Department of Anaesthesia, Southampton General Hospital, University Hospital Southampton NHS Foundation Trust, Tremona Road, Southampton SO16 6YD, UK; 2 NIHR Southampton Respiratory Biomedical Research Unit, Southampton, UK; 3 Department of Anaesthesia and Critical Care, University College London Hospitals, London, UK; 4 University College London, London, UK; 5 UCLH/UCL NIHR Comprehensive Biomedical Research Centre, London, UK

**Keywords:** Intravenous fluid, Critical illness, Surgery, Goal-directed therapy, Glycocalyx

## Abstract

Major surgery and critical illnesses such as sepsis and trauma all disturb normal physiological fluid handling. Intravenous fluid therapy for resuscitation and fluid maintenance is a central part of medical care during these conditions, yet the evidence base supporting practice in this area lacks answers to a number of important questions. Recent research developments include a refinement of our knowledge of the endothelial barrier structure and function and a focus on the potential harm that may be associated with intravenous fluid therapy. Here, we briefly describe the contemporary view of fluid physiology and how this may be disrupted by pathological processes. The important themes in critical illness fluid research are discussed, with a particular focus on two emerging ideas: firstly, that individualising fluid treatment to the patient, their underlying disease state and the phase of that illness may be key to improving clinical outcomes using fluid interventions and, secondly, that fluids should be considered to be drugs, with specific indications and contraindications, dose ranges and potential toxicities.

## Review

### Background

Disturbances of body fluid homeostasis are common in major surgery and a range of critical illnesses including sepsis and trauma. Administering appropriate intravenous fluid is a core part of medical care during these episodes. Despite years of research, there is still widespread debate about the best dosing strategy for these fluids and the optimum fluid composition for a given clinical situation. These are still important research questions, as there are clear signs from the current literature that fluid administration strategies have the power to affect clinical outcomes in a variety of areas.

Our study of fluid therapy is being refined by recent advances in basic physiology research. In particular, our knowledge of the structure of the vascular endothelium and the way in which fluid is handled at a capillary level has recently been redeveloped. There has also been progress in pharmacokinetic modelling of the movement of exogenous fluids between the body's fluid compartments. At the other end of the research spectrum, clinical fluid trials are becoming ever larger in an effort to address the limitations of the preceding literature, which is based predominantly on relatively small, single-centre studies.

Some key themes have emerged from this body of research. Firstly, it is increasingly clear that clinical context is key to the success or failure of a given fluid strategy. A fluid strategy which appears to reduce morbidity in one clinical population (e.g. patients undergoing major surgery) may actually be harmful to another (patients with established sepsis). Fluid therapies are being increasingly tailored to very tightly defined clinical subgroups and indeed to individual patients and the stage of their clinical course. A further theme is the emergence of toxicities related to fluid composition which have only become apparent through large clinical trials. Fluids are therefore increasingly being seen as drugs, with specific indications, contraindications and dose ranges [1].

In this review, we will outline the contemporary model of normal physiological fluid handling. The effects of a range of extreme physiological situations will be described, including acute haemorrhage and systemic inflammation. We will then review the latest literature in relation to clinical fluid strategies in a number of clinical settings Table 1.

### Fluid physiology

Water makes up approximately 60% of total body weight in the average adult. Due to the low water content of adipose tissue, this proportion varies widely with obesity (as low as 45%), age (higher in childhood, reducing to approximately 50% in the elderly) and sex (50% in an average adult female). Body water occupies functional and anatomical compartments. Fluid within cells—the intracellular compartment—is separated from the extracellular compartment by the cell membrane. This is impermeable to large hydrophilic molecules and charged particles, although these may cross it by specific transport mechanisms.

A proportion of extracellular water is contained within the bone and dense connective tissue, although due to slow kinetics this is viewed as non-functional. The extracellular compartment is further divided into interstitial fluid between cells and in lymphatics, intravascular fluid within blood vessels, and transcellular fluid including gastrointestinal secretions, joint fluid, cerebrospinal fluid and pleural, peritoneal and pericardial fluid.

**Figure 1:**
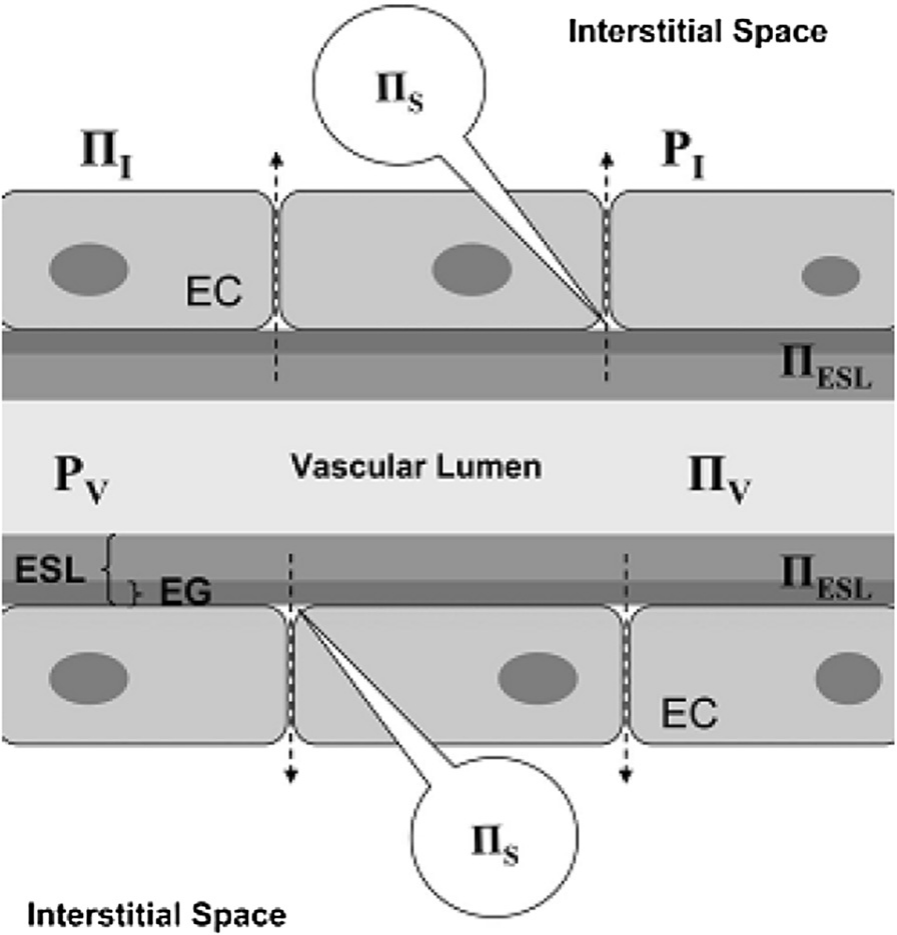
**The revised Starling principle and equation.** Hydrostatic pressures are higher within the vascular lumen (*P*_*v*_) than within the interstitium (*P*_*i*_), favouring outward fluid filtration. The endothelial surface layer (*ESL*) is formed by the endothelial glycocalyx (*EG*), which binds plasma proteins and excludes them from the subglycocalyx layer (*S*). This forms an oncotic gradient from the low protein concentration of the subglycocalyx (*IIs*) to the intravascular space (*IIv*). This gradient opposes outward fluid filtration. Net transcapillary flow (*J*_*v*_)—*dashed arrows*—can be expressed using the revised Starling equation: *J*_*v*_ = *K*_*f*_([*P*_*v*_ − *P*_*i*_] − *σ*[*π*_*v*_ − *π*_*s*_]), where *K*_*f*_ is the filtration coefficient and *σ* is the reflection coefficient (the degree to which the tendency of a macromolecule to cross the endothelial barrier is resisted). *EC*, endothelial cell. NB: Reproduced with permission from reference [2].

The vascular endothelium is the key barrier between the intravascular and interstitial spaces. It is particularly relevant clinically as it can be damaged during pathophysiological states such as inflammation, allowing potentially harmful fluid accumulation in the interstitial space. The vascular endothelium in a typical capillary is composed of a single layer of endothelial cells with intercellular clefts closed by tight junctions. The cells sit on a continuous basement membrane. On the vascular aspect of the endothelial cells lies a continuous layer of glycosaminoglycan chains, membrane-bound proteoglycans and glycoproteins. These form the endothelial glycocalyx layer (EGL), which is approximately 1 μm thick.Water and electrolytes can pass freely across the glycocalyx and then beyond the endothelial cells via the intercellular clefts. Proteins and other large molecules however are unable to pass through an intact glycocalyx and are transported in relatively small quantities across the endothelial cells by active processes. The EGL is therefore still part of the intravascular space but contains up to 700–1,000 ml of almost protein-free fluid with the same electrolyte composition as plasma. This creates a large protein concentration gradient—and oncotic pressure gradient—between the plasma and the space immediately below the EGL. This opposes the hydrostatic pressure gradient at the arteriolar end of capillaries, reducing the volume of water and electrolytes filtered out of the capillary. Net filtration across a capillary is expressed by the modified Starling equation (Figure 1).

### Pathophysiology of fluid homeostasis in critical illness

A wide variety of illnesses cause disturbances in fluid balance. This is usually due to inadequate or excessive volume in one or more functional fluid compartments, which may be associated with a disturbance in the barrier between those compartments.

Dehydration is a reduction in the water—and possibly sodium—content in all fluid compartments. It is a feature of excessive fluid loss through the skin (pyrexia, sweating), gastrointestinal tract (vomiting, diarrhoea, bowel obstruction) or kidneys (osmotic diuresis or polyuric renal failure) without adequate replacement.

Acute blood loss causes a sudden reduction in intravascular volume, cardiac filling and cardiac output. The transient reduction in arterial pressure is rapidly sensed by the high-pressure baroreceptors in the aortic arch and carotid sinus. The resultant sympathetic activation results in vasoconstriction, increased cardiac inotropy and increased heart rate. A range of other neurohumoral mechanisms are activated, including the renin-angiotensin-aldosterone axis and antidiuretic hormone release. These further contribute to vasoconstriction, reduction in glomerular filtration rate and renal retention of salt and water to compensate for the acute volume loss.

These early mechanisms may initially result in apparently normal haemodynamic observations. However, these responses divert blood away from ‘non-essential’ organs (kidney, gut, liver, skin) and towards essential organs (brain, heart). Healthy volunteer experiments have demonstrated signs of critical organ hypoperfusion, measured by a reduction in gut pH, despite normal-range blood pressure and heart rate after up to 30% blood volume loss [3]. This subclinical gut hypoperfusion may be a potent inflammatory stimulus, as reduced gut barrier function allows translocation of bacterial endotoxin into the systemic circulation [4,5].

Acutely reduced capillary pressure, seen in blood loss or due to widespread vasodilatation due to sepsis or anaesthesia, alters fluid movement at the capillary level also. In the initial phase, there may be an ‘autotransfusion’ of interstitial fluid into the intravascular space, although this is limited to approximately 500 ml [6]. Ongoing reduced capillary pressure reduces net filtration of fluid out of the intravascular space, and this state is maintained until capillary pressure returns to normal [6].

Fluid homeostasis becomes even more disturbed if the endothelium becomes damaged and loses its barrier function. A range of insults has been associated with degradation of the glycocalyx, including inflammatory mediators [7] or even natriuretic peptides associated with acute iatrogenic hypervolaemia [8]. Systemic inflammation also leads to an altered endothelial cell phenotype and increased endothelial pore size. This endothelial dysfunction allows the loss of proteins into the interstitial space, accompanied by excess water. This overloads the lymphatic system and results in oedema within the compliant tissues such as connective tissue, lungs and gut. The loss of plasma proteins and oncotic pressure also aggravates excessive capillary filtration, causing hypovolaemia and organ dysfunction.

The clinical management of intravenous fluid administration therefore has the potential to influence organ function, morbidity and mortality in pathophysiological settings. If fluid replacement is inadequate during critical illness, inadequate organ blood flow and therefore inadequate delivery of oxygen and energy substrates may occur. This results in inefficient anaerobic cellular metabolism and in severe cases cell death and organ failure. Conversely, excessive salt and water administration, particularly in the setting of endothelial dysfunction, may worsen organ oxygenation and function through oedema formation.

Again, the capillary handling of administered intravenous fluids is critical and has recently been re-examined. Free water contained in hypotonic solutions distributes evenly throughout all fluid compartments, leaving only a small proportion within the intravascular space. Traditionally, isotonic electrolyte solutions were thought to distribute evenly through the extracellular (intravascular and interstitial) space only, leaving approximately 20%–25% of their volume in the intravascular space. Colloids, due to their superior oncotic pressure, were thought to initially remain almost completely within the intravascular space and were seen as the best fluid for acute plasma volume expansion.

It is now proposed that the ‘context’ of capillary pressure is important [2]. At low capillary pressures, infusion of colloid will expand the plasma volume, but the large molecules should not pass beyond an intact glycocalyx. Crystalloid infusions will expand the entire intravascular volume, i.e. both plasma and EGL. In both cases, net capillary filtration will remain close to zero until capillary hydrostatic pressure becomes normal or supra-normal. This may help to explain the observation in numerous clinical studies that similar resuscitation end points can be achieved using 50% more crystalloid than colloid, rather than the much larger crystalloid volumes traditionally predicted [9-11]. Pharmacokinetic studies have also demonstrated that in anaesthetised patients—with presumably low capillary pressure—around 60% of an isotonic crystalloid solution is retained in the circulation during the infusion, as redistribution to other compartments takes 20–25 min [12].

For both fluid types, in the setting of normal or supra-normal capillary pressure, net filtration will occur, with loss of fluid from the intravascular space and the potential for oedema formation. Although in health the maintained oncotic pressure associated with colloids helps to limit this filtration, this effect is reduced in the presence of endothelial dysfunction. Here, there is the potential for large colloid molecules to pass freely into the interstitial space. However, as more crystalloid volume is needed to achieve similar resuscitation endpoints to colloids, patients resuscitated with crystalloid do ultimately gain a more positive fluid balance [13] Table 1.

**Table 1:** Fluid therapy terminology

**Term**	**Summary**		
**Osmotic and oncotic pressure**	— osmotic pressure is the hydrostatic pressure that would be required to resist the diffusion of water across a semipermeable membrane from a higher solute concentration to a lower solute concentration. Oncotic pressure is the portion of osmotic pressure which is due to large molecular weight particles, particularly proteins.		
**Fluid tonicity**	— the effective osmolality of a solution in relation to a specific semipermeable membrane and therefore a useful way of describing a given fluid's in vivo behaviour. For example, although 5% dextrose has a similar *ex vivo* osmolality to 0.9% sodium chloride, after infusion the dextrose is taken up into cells, rendering the solution effectively hypo-osmolar with respect to the cell membrane, i.e. hypotonic; 0.9% sodium chloride remains isotonic due to the retention of sodium and chloride ions in the extracellular space.		
**Crystalloid**	— solutions of glucose and/or electrolytes in water.		
**Colloid**	— a dispersion of large molecules or ultramicroscopic non-crystalline particles in a carrier crystalloid. It includes gelatins, starches and dextrans.		
**Balanced solutions**	— those with a composition more similar to plasma than to 0.9% sodium chloride. It is achieved by replacing a proportion of the chloride with stable organic anionic buffers such as lactate, gluconate or acetate.		
**Goal-directed haemodynamic therapy**	— the use of cardiac output monitoring to guide fluid and inotrope therapy. Key physiological goals are targeted in specific treatment algorithms. This may be a predefined increase in global oxygen delivery or stepwise increases to wards a maximal cardiac stroke volume. This treatment—as compared with fluid dosing based on clinical assessment or ‘per weight’ basis—has been used in various forms for over 40 years. Early variants were guided by the pulmonary artery catheter, but several minimally invasive devices are now in use.		

### Therapeutic fluid strategies and clinical outcome

As a core part of medical treatment during acute illness and trauma, it is not surprising that a number of fluid administration strategies have been trialled in various clinical settings over the years. This evidence base has shown that fluid strategies have the potential to influence morbidity and possibly mortality outcomes after critical illness and major surgery. However, clear-cut answers which can be confidently translated to widespread clinical practice are rare. A summary of some of the critical illness fluid research areas is contained in Table 2.

**Table 2:** Key themes in critical illness fluid research

**Patient group**	**Research question**	**Early trial evidence**	**Subsequent developments and ongoing research questions**
Sepsis	Does early goal-directed bolus fluid therapy improve survival from severe sepsis?	Key single-centre study by Rivers showing reduced mortality when bolus fluid therapy targeting central venous pressure and mixed venous saturations was used at presentation of septic patients to hospital [14].	Further developments in sepsis care through treatment ‘bundles’ and the need to test the intervention have led to a re-appraisal of the benefits of this intervention. The ProCESS trial recently published comparing protocol-driven goal-directed therapy with protocol (but not goal-directed) therapy and with standard care in 1,341 patients. No differences in mortality at 60, 90 or 365 days [15]. Further multicentre trials based on River's original protocol are nearing completion in the UK (ProMISe) and Australasia (ARiSE).
Mixed critical care populations	Is fluid composition used for resuscitation associated with acute kidney injury and increased mortality?	Early starch-based colloids with high molecular weight were associated with renal dysfunction [16]. Lower molecular weight starches were brought to market based on efficacy data but without trials large enough to detect possible harm.	A number of large trials have demonstrated an increase in the need for renal replacement therapy with modern starches, when compared to isotonic crystalloids. This has been shown in both septic [17,18] and mixed critical care [13] populations. Despite concerns over trial methods, for example that some patients had been partially resuscitated before trial entry, use of starches has now been restricted (USA, Europe) or stopped (UK). A recent network meta-analysis has suggested that renal replacement requirement is highest in association with starches, followed by isotonic saline then balanced crystalloids [19]*.*
Surgery	Does goal-directed haemodynamic therapy improve outcomes for patients undergoing major surgery?	Survivors of high-risk surgery were found to achieve higher global oxygen delivery levels than non-survivors. These ‘survivor values’ were then used as therapeutic targets in subsequent interventional trials, with benefit shown [20,21].	Reduced popularity of the pulmonary artery catheter followed by minimally invasive cardiac output monitors and an evolution of fluid administration protocols. Single-centre trials from the 1990s onwards showed reduction in hospital length of stay and postoperative morbidity in goal-directed therapy intervention groups. A recent Cochrane review points to no harm and probably morbidity benefit from goal-directed therapy, although studies span over 30 years of trials [22]. A large contemporary multicentre trial also involving an inotrope in the intervention group (OPTIMISE) again suggested benefit but lacked statistical significance. The accompanying update to the meta-analysis strengthens the evidence for a reduction in postoperative morbidity [23]. Studies in emergency surgery are lacking.
Surgery	Can targeting a fluid ‘dose’ lead to improved outcomes in patients undergoing major surgery?	Patients receiving ‘standard’ volumes of fluid in the peri-operative phase were shown to have more postoperative morbidity than those receiving ‘restrictive’ volumes [24].	This research theme has been hampered by varying definitions of what constitutes liberal and restrictive volumes of fluid. Subsequent trials have therefore shown contrasting results. A common theme though is the association between a positive fluid balance in the immediate peri-operative period (>3,500–5,000 ml) and worse postoperative outcomes. Giving larger volumes of fluid without robust physiological monitoring appears to be associated with worse outcomes, even when overall quantities given may be similar to those used in goal-directed therapy interventions [25]. A large and hopefully definitive multicentre trial (RELIEF) is underway in Australasia and the UK.
Surgery	Does isotonic saline cause harm to patients undergoing surgery when compared with balanced solutions?	Concerns that isotonic saline is unphysiological in composition and may cause harm through reduction in renal blood flow and hyperchloraemic acidosis. These have never been backed up by trials with adequate statistical power.	Observational studies suggest harm with saline as compared to balanced solutions [26,27]. A Cochrane review suggests an increase in postoperative acidosis and need for compensatory hyperventilation with saline used in the peri-operative setting [28]. No increase in postoperative renal morbidity. However, there is a lack of high-quality large trials, and this remains an important topic for ongoing research.
Trauma	Does limiting volumes of early resuscitation fluid improve outcomes from trauma?	Early suggestions from battlefield that fluid resuscitation may be harmful in ‘wound shock’ [29]. Renewed interest following pre-hospital randomised trial in penetrating trauma showing reduced mortality in limited vs. standard resuscitation [30].	Permissive hypotension with limited volumes of clear fluid given prior to achieving haemostasis is proposed as a part of ‘damage control resuscitation’ [31]. This also includes early surgical or radiological control of haemorrhage and steps to limit coagulopathy, including tranexamic acid and high ratios of plasma and platelets to red blood cell units transfused [32]. Controversy remains about the place of limited volume early resuscitation, as follow-up trials have not shown in-hospital or 30-day mortality benefit when blunt trauma patients are included [33,34]. Hypotension may also worsen co-existing brain injury [35].
Trauma	Does hypertonic saline improve outcomes from trauma?	Hypertonic solutions may have the ability to draw water from the intracellular to the extracellular compartment, achieving plasma volume expansion with minimal volume of fluid administered.	Limited human studies examining hypertonic saline used early in trauma resuscitation. One large trial stopped early as interim analysis demonstrated futility [36]. Hypertonic solutions not routinely recommended in trauma resuscitation. Although hypertonic saline is effective at reducing raised intracranial pressure [37], there is no benefit in early traumatic brain injury when intracranial pressure is not monitored [38].

There are a number of challenges which have hampered evidence-based practice in this area. Firstly, fluid therapy is a prime example of what the Medical Research Council terms a ‘complex intervention’ [39]. This complexity lies at multiple levels, from the biological interaction between administered fluid and the pathophysiological setting, through the interaction of the clinician with choice of fluid, monitoring and therapeutic strategy, to clinician beliefs, resource limitations, health-care systems, guidelines and financial incentives which may promote or inhibit the adoption of certain fluid interventions.

One example of this complexity leading to ongoing uncertainty is in fluid strategies around the time of major surgery. An approach to haemodynamic therapy, based on targeting supra-normal global oxygen delivery—monitored using the pulmonary artery catheter—was initially investigated in the 1970s [20,40]. Despite early promise, controversy developed over the safety of pulmonary artery catheters though a number of clinical trials [41]. By the 1990s, they had fallen out of widespread use. New goal-directed haemodynamic therapy strategies using less invasive technologies such as the transoesophageal Doppler monitor were therefore developed and tested. However, the evidence of benefit was accrued slowly through a number of single-centre trials, and it was not until 2011 that the National Institute for Clinical Excellence recommended their use in UK peri-operative practice [42]. Despite this, widespread uptake into clinical practice was limited. Financial incentives through ‘quality payments’ have been used in an attempt to promote the technology in the UK. However, in the intervening decades, changes in surgical practices and care pathways have developed, reducing baseline morbidity and mortality. Despite nearly 40 years of research, it therefore remains uncertain whether goal-directed haemodynamic therapy is beneficial for contemporary surgical populations. Increasingly large-scale trials are currently in progress or development in order to provide a definitive answer to this question.

Even at a basic physiological, pathological and pharmacological level, there is considerable complexity which may be important in fluid therapy. The mechanisms of action of fluid strategies at an organ and cellular level have been relatively under-explored in relevant human clinical settings. This gap in the knowledge base may help to explain the variable success of many fluid clinical trials. The ultimate goals of fluid therapy are to provide enough circulating volume and blood flow to organs, so that the quantities of oxygen and nutrients delivered are sufficient to meet their metabolic requirements, while avoiding iatrogenic harm through excess dosing of water, electrolytes or exogenous compounds. Yet this apparent simplicity belies the biological complexity of achieving these goals in diverse disease settings. The clinical benefit or harm brought by administering fluids may result from fluid composition, dose, physiological target, timing during the illness, or complex interactions between these factors, the patient's pre-morbid phenotype and the acute but ever-changing pathophysiological processes.

Developing the evidence base behind critical illness fluid practices further therefore needs a combination of basic science, translational trials and clinical trials. Controlled trials with large sample sizes are necessary to definitively detect differences in outcome which may be small but important when applied to the huge number of patients undergoing major surgery or critical illness. But achieving this size often requires pragmatic trial design and heterogeneous groups (e.g. ‘all major surgical patients’ or ‘all critical care patients’) which potentially obscure the nuances of which individual patients might get benefit or harm and when. More mechanistic studies are needed to explore what aspect(s) of the intervention strategy brought the benefit or harm and to address apparently basic but as-yet unanswered questions, such as how the body handles the currently available range of exogenously administered anions.

### Individualisation of fluid strategies

It is implausible that one fluid administration strategy will ever be beneficial to all patients in all clinical settings at all time points in their critical illness or physiological insult. There is an increasing realisation that trial findings in one group cannot be readily extrapolated to other groups. The theme of ‘individualisation’—tailoring fluid interventions to each patient and each setting—is gaining momentum and is seen in the following areas.

#### Patient: tailoring to patient physiology

Goal-directed haemodynamic therapy in patients undergoing major surgery is a longstanding example of adapting fluid dosing to a patient's baseline physiology. This is also true of contemporary algorithms aimed at optimising cardiac stroke volume. These involve a physiological ‘test’ in the form of a fluid bolus with ongoing monitoring of stroke volume. If the patient is fluid replete—or cardiac performance is beyond the optimum point of the Starling curve—there will be no further increase in stroke volume and fluid bolus administration can stop. This is in contrast to other dosing strategies based on patient weight or physiological signs which are poor at predicting a response to fluid.

#### Timing: tailoring the fluid strategy to the pathophysiological phase

There is growing evidence that the timing of plasma volume expansion is important in its potential success. This may be seen even within the relatively short time frame of a surgical operation. One study showed that despite only modest differences in the overall volume of fluid given to patients undergoing surgery, the use of cardiac output monitoring to guide fluid input led to fluid being given earlier in the operation, which led to sustained increases in cardiac output not seen in the control group [43]. This was associated with a reduction in postoperative morbidity. More broadly, initial studies pointed to potential benefits of goal-directed fluid resuscitation early in severe sepsis [14], whereas later aggressive fluid administration during established critical illness appears to be harmful [44-46]. Differences in the time of entry into clinical trials of fluid resuscitation may also explain differing messages about potential harm from colloids used for volume expansion [13,47].

The differing physiological requirements from fluid therapy during the developing stages of critical illness and circulatory shock have been recognised in a new proposed treatment approach [48]:

• Salvage phase—the immediate phase where fluid resuscitation is used as a lifesaving measure, guided by immediately available information such as clinical assessment, blood pressure and heart rate

• Optimisation phase—guided by more in-depth monitoring, and aiming to optimise global oxygen delivery, with resultant improvements in arterial lactate and mixed venous blood desaturation

• Stabilisation phase—a more cautious approach to fluid administration, aiming to minimise iatrogenic harm caused by fluid overload and institute more general organ support where required

• De-escalation phase—appropriate during the start of resolution of critical illness, aiming to reduce vasoactive treatments and achieve a negative fluid balance

#### Disease: tailoring fluid strategy to the underlying pathophysiology

A number of clinical syndromes (‘critical illness’ or ‘circulatory shock’) appear to have common features which may be addressed by similar fluid approaches. Yet the underlying disease process seems to be critical to the benefit or harm brought by these fluid strategies. In mixed critical care populations, including severe sepsis, there is growing evidence that starch-based colloids are associated with an increased requirement for renal replacement therapy [17,18]. This has led to restrictions on the licences for these products. In contrast, many of the trials showing the benefits of goal-directed haemodynamic therapy in major surgical patients have used colloids—including starches—for their intervention fluid boluses.

Another example of this is the Saline vs. Albumin Fluid Evaluation (SAFE) study, a large multicentre trial comparing albumin with isotonic saline for fluid resuscitation in a mixed critical illness population [49]. Overall no difference in outcome was found, but importantly predefined subgroup analyses highlighted clinical populations worthy of further investigation. Patients with brain injury appeared to have worse outcomes in the albumin arm. It has been suggested that this was due to the slight hypotonicity of the albumin preparation used (260 mOsm.L^−1^), which may have contributed to intracranial hypertension [50]. Conversely, patients with sepsis seemed to have better outcomes with albumin. This led to the recently published ALBIOS trial of albumin supplementation in patients with sepsis, powered specifically to detect mortality difference in this more homogeneous group [51]. No differences in the primary outcome were found, and despite differences in protocol from the original SAFE study, this trial demonstrates the utility of focussing on more tightly defined disease phenotypes even in large randomised trials.

Lastly, the most basic example of matching fluid input to the pathophysiological situation has been summarised in the UK national guidance on intravenous fluid use [52]. Here, clinicians are encouraged to consider exactly why they are giving a fluid in a particular situation and match the fluid composition and volume to one of the following physiological needs:

• Resuscitation (bolus therapy recommended, using fluids with sodium in the range 130–154 mmol.L^−1^ but avoiding starches)

• Routine maintenance (25–30 ml.kg^−1^.day^−1^ with 1 mmol.kg^−1^.day^−1^ each of sodium, potassium and chloride, plus 50–100 g.day^−1^ of glucose)

• Replacement due to ongoing losses and redistribution (fluid composition tailored to match the estimated water and electrolyte loss)

#### Health-care setting: tailoring fluid strategy to the wider clinical context

A further layer of complexity in fluid treatments may come from the health-care setting in which they are used. Fascinating insights have come from the FEAST study, which took the principle of fluid bolus therapy early in the presentation of patients with sepsis but applied it in the setting of children presenting to hospital in Africa [53]. In contrast to the potential benefits found in previous studies, in this population there was a 3.3% absolute increase in mortality at 48 h in the fluid intervention group. Subsequent analysis has suggested that these excess deaths were probably not attributable directly to fluid overload (e.g. through respiratory or neurological events) [54]. Instead, the bolus fluid group had an initial improvement in haemodynamic parameters, but then went on to have an excess of cardiogenic or shock-related terminal events. One interpretation of this is that early large-volume fluid resuscitation sets up a series of adverse processes, including vascular dysfunction and impaired myocardial performance. Thus, the rapid reversal of shock may then lead to a requirement for inotropic and other systems support, which are hard to deliver in health-care systems without intensive care facilities.

## Conclusions

Abnormal fluid handling is a central part of many critical illnesses, and exogenous fluid administration has become a core part of medical care in these settings. Despite apparently rational physiological goals, the reality of successful fluid intervention is extremely complex at a basic biological, clinical and health-care system level. As a result, many of the key research questions in this field have not yet been definitively answered despite years of research. Large trials and meta-analyses have raised the possibility of fluid-related toxicities long after these fluids have been brought to market. Intravenous fluids must be re-framed as drugs, with indications, contraindications, side effects and dose ranges. Lastly, the emergence of the theme of individualisation—to timing, patient, disease state and health-care setting—is an important step forward, but a combination of robust basic science, translational trials and large clinical trials will be required to progress this area further.

## Abbreviations

EG: endothelial glycocalyx; EGL: endothelial glycocalyx layer; ESL: endothelial surface layer; IIs: subglycocalyx oncotic pressure; IIv: intravascular oncotic pressure; *J*_*v*_: net transcapillary flow; *P*_*i*_: interstitial hydrostatic pressure; *P*_*v*_: vascular lumen hydrostatic pressure; S: subglycocalyx layer.

## Competing interests

MRE declares that he has no competing interests. MGM has received honoraria for speaking, or consultation and/or travel expenses from Baxter, B. Braun, Covidien, Fresenius Kabi, Hospira and LiDCO. He is the National Clinical Advisor for the Department of Health Enhanced Recovery Partnership, is a Smiths Medical Professor of Anaesthesia and Critical Care at UCL, has consulted AQIX (start-up company with a novel crystalloid solution—pre-clinical), is the Director of Medical Defence Technologies LLC (‘Gastrostim’ patented) and is a co-inventor of ‘QUENCH’ (pump) IP being exploited by UCL Business. Professor Mythen's institution has also received charitable donations and grants from Smiths Medical Endowment and Deltex Medical. Professor Mythen is also a co-author of the GIFTASUP guidelines on peri-operative fluid management, the Chair of the National Institute of Academic Anaesthesia, a Board member of the Faculty of Intensive Care Medicine, a Council member of the Royal College of Anaesthetists of Great Britain and Ireland, the Editor in Chief of Peri-operative Medicine, on the Editorial Board of the BJA and Critical Care, a member of the Improving Surgical Outcomes Group, a member of the NICE IV Fluids Guideline Development Group (now an expert advisor) and the Co-Director of Xtreme Everest.

## Authors’ contributions

MRE and MGM both conceived and planned this review. MRE drafted the manuscript. MGM helped to draft the manuscript and revised it critically. Both authors read and approved the final manuscript.
